# Hypertension Treatment Cascade in Rural Delhi: Prevalence, Awareness, Treatment, and Control Among Adults

**DOI:** 10.7759/cureus.105597

**Published:** 2026-03-21

**Authors:** Swaleha Furqan, Geeta Yadav

**Affiliations:** 1 Community Medicine, Vardhman Mahavir Medical College and Safdarjung Hospital, New Delhi, IND; 2 Epidemiology and Public Health, Vardhman Mahavir Medical College and Safdarjung Hospital, New Delhi, IND

**Keywords:** awareness, control, hypertension, prevalence, risk factors, treatment

## Abstract

Introduction: A majority of patients with hypertension remain undiagnosed, untreated, non-compliant, and poorly controlled at every stage. However, there is a paucity of information on the hypertension treatment cascade, especially among the population in rural areas. The aim of this study was to find out the prevalence of different stages of the hypertension treatment cascade among the rural adult population of Delhi.

Methods: A community-based cross-sectional study was conducted among 325 adult participants in a rural area of Delhi, using a pre-tested, pre-coded, semi-structured, interviewer-administered questionnaire. Blood pressure was measured on two separate occasions, one week apart.

Results: The overall prevalence of hypertension was 116 (35.7% (95% CI: 30.5-40.9)). Male participants had a higher prevalence of hypertension than female participants. Among the 116 participants identified as hypertensive, 71 (61.2%) were aware of their hypertensive status. Of these, 52 individuals (44.8% of all hypertensives) reported being on treatment. Blood pressure control was achieved in 17 individuals (14.6% of all hypertensives). Non-compliance with treatment was common, with financial constraints (10; 19.2%) and fear of side effects (7; 3.4%). Significant factors associated with hypertension included age, time spent sitting/reclining, tobacco use, comorbidity, and waist circumference (p < 0.05).

Conclusion: The present study revealed a high burden of hypertension among adults in rural Delhi. Although the rate of awareness was relatively high, the rates of treatment and control were far from desirable. Compliance with medication and lifestyle interventions was also poor. Strengthening community screening and primary care services is necessary for early detection, continuous management, and improved health outcomes.

## Introduction

Hypertension is a major public health challenge globally and in India. Previously considered a condition predominantly affecting affluent populations, hypertension is now widely prevalent across all age groups, socioeconomic strata, and geographic regions. It is a leading contributor to non-communicable disease (NCD)-related mortality worldwide and ranks as the third most important risk factor for years of life lost due to morbidity and premature death [1,2].

In 2019, globally, approximately 33% of adults aged 30-79 years were living with hypertension [3]. Between 1990 and 2019, the number of individuals with hypertension increased from an estimated 650 million to 1.3 billion [4]. Despite advances in screening and management, substantial gaps persist in awareness, treatment, and control, particularly in low- and middle-income countries. Worldwide, nearly 46% of adults with hypertension are unaware of their condition, only 42% receive treatment, and approximately 21% achieve adequate blood pressure control [5].

In India, the burden of hypertension remains high and continues to rise. According to the World Health Organization (WHO), the prevalence of hypertension among adults aged 30-79 years was approximately 31% in 2019 [3]. Data from the National Family Health Survey-5 (NFHS-5) indicate that 24% of men and 21.3% of women aged 15 years and above have hypertension [6]. Although men demonstrate a higher prevalence, treatment and control rates vary by sex. Among individuals with hypertension, only 30% receive treatment, including 25% of men and 35% of women. Furthermore, adequate blood pressure control is achieved in only 15% of treated individuals, with lower control rates observed among women [6].

Recent evidence from rural India highlights a concerning increase in hypertension prevalence. A repeated cross-sectional study conducted in the National Capital Region reported that the prevalence increased from 11.2% to 28.9% in rural populations over the past 20 years (1991-2012) [7]. Another large community-based study conducted across 300 rural and 300 urban ward clusters reported a hypertension prevalence of 34% in urban areas and 25.7% in rural areas [8]. Hypertension substantially increases the risk of stroke, ischemic heart disease, heart failure, chronic kidney disease, and other cardiovascular complications, thereby contributing significantly to morbidity and mortality [3,4].

To address this growing burden, India aligned with the global “25 by 25” target, which aimed to reduce premature mortality from NCDs by 25% by the year 2025. Among the nine voluntary global targets, one specifically focuses on achieving a 25% relative reduction in the prevalence of raised blood pressure [9].

Given the gaps in hypertension awareness, treatment, and control, particularly in rural settings, this study aimed to determine the prevalence, awareness, treatment, and control of hypertension among adults in a rural area of Delhi and to identify factors associated with each stage of the hypertension treatment cascade.

## Materials and methods

Study population and sample size

This community-based cross-sectional study was carried out from March 2023 to August 2024 among adults aged 18 and above residing in villages under the Urban Primary Health Centre (UPHC), Fatehpur Beri, New Delhi. It is an area falling under the Hauz Khas subdivision of the South West Delhi District.

For the sample size calculation, the prevalence of hypertension among adults in the rural population was taken as 25.7% based on the study done by Amarchand et al. [8]. Using a 95% confidence interval, the prevalence of 25.7%, and a 5% absolute precision level, the sample size was calculated by the formula, (Z_α/2_)^2^ x P x Q/L^2^. The sample came out to be 295, and after adding a 10% non-response rate, the final sample size was estimated to be 325.

Sampling and data collection

A total of 11 areas come under UPHC Fatehpur Beri. According to the 2011 census data, six areas have been mentioned as census towns. Thus, five villages (Bara Bans, Chota Bans, Sahoorpur, Kharak, and Satbari) were considered for selecting the study population. The sample size was allocated to each village using the probability proportional to size (PPS) method, based on population data from Census 2011.

In each village, the investigator started by standing at the village's centre and spinning a bottle on the floor; in the direction in which it pointed, the first household in that direction was selected, and subsequent households were visited consecutively until the required sample size for that village was achieved. Each consecutive house was visited, and one person aged 18 and above in the house was selected if they met the inclusion criteria. In households with more than one eligible adult (≥18 years), one participant was selected using simple random selection (draw of lots) to avoid selection bias. If a household was locked or no eligible participant was available after two visits, the next adjacent household was approached. A pre-designed, semi-structured, interviewer-administered questionnaire developed by the author was used to collect relevant data (see the Appendices).

Blood pressure was measured using a calibrated aneroid sphygmomanometer with an appropriate cuff size. Participants were asked to rest for at least five minutes in a seated position, with their back supported and feet flat on the floor, before measurement. Blood pressure was measured on the right arm supported at heart level. Three readings were taken at an interval of one to two minutes; the first reading was discarded, and the average of the remaining two readings was used for analysis. To improve accuracy, blood pressure measurements were obtained on two separate occasions at least one week apart, and the average value was used in the final analysis.

Operational definitions were as follows: systolic blood pressure (SBP) 120-139 mmHg and/or diastolic blood pressure (DBP) 80-89 mmHg for prehypertension and SBP ≥140 mmHg and/or DBP ≥90 mmHg or on blood pressure-lowering medication for hypertension. Those who were diagnosed with hypertension were further classified into stage I (SBP 140-159 mmHg and/or DBP 90-99 mmHg) and stage II (SBP ≥160 mmHg and/or DBP ≥100 mmHg) [10].

Those who knew about their diseased status before the data collection period were considered as aware. Those who were on allopathic pharmacological treatment during data collection were considered as on treatment and those having systolic blood pressure <140 mmHg and diastolic blood pressure <90 mmHg were labelled as controlled.

Inclusion/exclusion criteria

All adults aged ≥18 years residing in the selected village were included. Pregnant women and women in the postpartum period were excluded from the study.

Ethical considerations

The study was conducted after obtaining approval from the Institutional Ethics Committee of Vardhman Mahavir Medical College and Safdarjung Hospital, New Delhi. Written informed consent was obtained from all participants before enrolment in the study. Participants were informed about the purpose of the study, and their participation was entirely voluntary. Confidentiality and privacy of the participants were strictly maintained.

Statistical analysis

Data were entered in Microsoft Excel (Microsoft Corporation, Redmond, USA) and were cleaned for errors and missing values. Data analysis was done using IBM SPSS Statistics, version 21.0 (IBM Corp., Armonk, USA). Qualitative data were analysed using a chi-square/Fisher exact test. Statistical significance was defined as p < 0.05. Bivariate and multivariate logistic analyses were carried out to determine the factors associated with hypertension.

## Results

The mean age of study participants was 40.4 ± 15.4 years, and the majority (233, 71.7%) were women; 167 (51.4%) were Hindus, and 155 (47.7%) were Muslims. Nearly one-third of the study participants belonged to the lower middle class, 183 (56.3%) belonged to a nuclear family, 114 (35.1%) had no formal education, and only 95 (29.2%) were gainfully employed at the time of study (Table 1).

**Table 1:** Socio-demographic characteristics (N=325)

Characteristics	Number (n)	Percentage
Age (in complete years)		
<30	92	28.3
30-59	178	54.8
>60	55	16.9
Gender		
Female	233	71.7
Male	92	28.3
Religion		
Hinduism	167	51.4
Islam	155	47.7
Sikh	3	0.9
Educational status		
Illiterate	114	35.1
Primary and middle school	144	44.3
Senior secondary and above	67	20.6
Marital status		
Married	258	79.4
Unmarried	40	12.3
Widow/widower	27	8.3
Type of family		
Joint	142	43.7
Nuclear	183	56.3
Occupational status		
Homemaker	196	60.3
Student/unemployed/retired	34	10.5
Gainfully employed	95	29.2
Income (Rs)		
>8480 (upper class)	30	9.2
4240–8480 (upper middle class)	84	25.8
2544–4240 (middle)	94	28.9
1272–2543 (lower middle)	110	33.8
<1272 (lower)	7	2.2

Hypertension treatment cascade (prevalence, awareness, treatment, and control)

Out of 325 participants, 118 (36.3%) had never had their BP measured; among those who had their BP measured, 165 (79.7%) had it measured within the last six months. The mean systolic BP was 124.9 mmHg with a standard deviation (SD) of 17.8, and the mean diastolic BP was 82.6 ± 10.9 mmHg. Prehypertension was present in 106 (32.6%) participants. The overall prevalence of hypertension was 116 (35.7%). Among those identified as hypertensive (n = 116), 32 (27.6%) were aged 60 years and above, 31 (26.7%) were in the 50-59 year age group, 22 (19.0%) were aged 40-49 years, 19 (16.4%) were in the age group of 30-39 years, and 12 (10.3%) were aged below 30 years.

**Figure 1:**
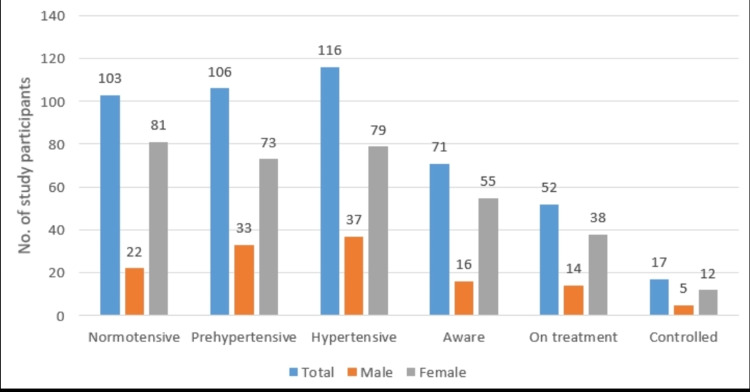
Hypertension treatment cascade

Out of 116 hypertensive participants, 71 (61.2%) were aware of their condition, and 52 (44.8%) were on treatment. Awareness about hypertensive status was higher among female participants as compared to male participants. About 52 (73%) of the aware hypertensive participants reported taking medications for hypertension ever in their life, and the remaining 19 (26.8%) participants never started treatment. Thus, the overall proportion of hypertensives on treatment at the time of study was 52 (44.8%). The proportion of female participants on treatment was higher than that of male participants. Of the total hypertensives, only 17 (14.6%) had their BP under control, whereas among those on treatment, the proportion was 32.6% (17 out of 52). The rate of control was proportionately better among female participants (Figure 1).

Of the participants on treatment, 22 (42.3%) utilized both private and government facilities for treatment, whereas 17 (32.7%) used only government health facilities. The majority of them (45, 86.5%) were utilizing only the allopathic system, and the rest were using other systems of medicine. About 53.3% (24) of participants admitted to occasionally forgetting to take their medicines, while 21 (46.7%) were either taking them irregularly or intentionally stopped taking them. Within the last two weeks, 6 (13.3%) participants reported missing taking their medication 10-14 times, 11 (24.4%) missed taking 5-9 times, and the majority (28, 62.3%) of participants forgot taking medicines 0-4 times (Table 2).

**Table 2:** Distribution of study participants according to compliance with treatment of hypertension in the last two weeks

Compliance	Number (n)	Percentage
Medicine is taken as prescribed by the doctor (n=52)		
Yes	45	86.5
No	07	13.5
Usually forgets to take medicine (n=45)		
Yes	24	53.3
No	21	46.7
In the last 14 days, missed taking medication (n=45)		
0-4 times	28	62.3
5-9 times	11	24.4
10-14 times	6	13.3

Regarding lifestyle modification, 42 (59.2%) participants reported reducing their salt intake, 30 (42.3%) decreased fat intake, whereas a mere 13 (18.3%) reported engaging in physical activity.

Financial constraints (10, 22.2%), fear of side effects (7, 15.5%), and not feeling like taking medication (7, 15.5%) were important reasons reported for non-compliance. Non-availability of medicine (6, 13.3%), time consumed in visiting healthcare facilities (6, 13.3%), healthcare facilities being far away (4, 8.8%), too many pills to take (4, 8.8%), no one being there to accompany one to the health facility (3, 6.6%), and busy schedule (3, 6.6%) were some of the other common reasons reported for non-compliance. It is important to note that these reasons are not mutually exclusive, indicating that participants may have experienced multiple barriers to treatment compliance (Table 3).

**Table 3:** Distribution of study participants according to reasons for non-compliance with the treatment of hypertension (n=45) Reasons are not mutually exclusive.

Reason	Number (n)	Percentage
Financial constraint	10	22.2
Do not feel like taking medicine	7	15.5
Fear of side effects	7	15.5
Non-availability of medicine	6	13.3
Too much time is consumed at healthcare facilities	6	13.3
Healthcare facilities are too far away	4	8.8
Too many pills	4	8.8
No one is there to accompany one to the health facility	3	6.6
Busy schedule	3	6.6

Associated risk factors

Multivariable logistic regression analyses, age, time spent sitting/reclining, tobacco use, comorbidity, and waist circumference were significantly associated with hypertension. Participants aged ≥30 years had higher odds of hypertension compared with those aged <30 years (AOR: 2.86). Participants spending less than nine hours a day sitting or reclining had lower odds of hypertension than those spending nine or more hours a day (AOR: 0.27). Non-tobacco users had lower odds of hypertension compared with tobacco users (AOR: 0.38). Participants without a comorbidity had lower odds of hypertension compared with those with a comorbidity (AOR: 0.37). Increased waist circumference was independently associated with higher odds of hypertension (AOR: 3.16) (Table 4).

**Table 4:** Multivariable logistic regression for associated factors for hypertension (N=325) p-value <0.05 was considered significant. Variables with p < 0.20 in bivariate logistic regression, along with biologically plausible variables, were included in the multivariable logistic regression model.

Variable	Category	Unadjusted OR (95% CI)	p-value	Adjusted OR (95% CI)	p-value
Age	<30 years	1	—	1	—
	≥30 years	5.22 (3.16–8.61)	<0.01	2.86 (1.51–5.42)	<0.01
Time spent sitting/reclining	≥9 hours/day	1	—	1	—
	<9 hours/day	0.13 (0.05–0.30)	<0.01	0.27 (0.10–0.71)	<0.01
Tobacco use	Yes	1	—	1	—
	No	0.34 (0.20–0.61)	<0.01	0.38 (0.19–0.77)	<0.01
Comorbidity	Yes	1	—	1	—
	No	0.22 (0.11–0.44)	<0.01	0.37 (0.16–0.84)	0.01
Waist circumference	<90 cm in males, <80 cm in females	1	—	1	—
	≥90 cm in males, ≥80 cm in females	2.79 (1.72–4.52)	<0.01	3.16 (1.40–7.17)	<0.01

Multivariable logistic regression analysis showed that awareness of hypertension was significantly associated with age, education, sitting time, comorbidity, and waist circumference. Participants aged ≥30 years had lower odds of being aware of their hypertensive status compared with those aged <30 years (AOR: 0.336). Literate participants had higher odds of awareness than illiterate participants (AOR: 2.559). Participants with sitting time less than nine hours a day had higher odds of awareness compared with those with sitting time more than nine hours a day (AOR: 3.61). Absence of comorbidity was associated with lower odds of awareness compared with the presence of comorbidity (AOR: 0.313), and abnormal waist circumference was also associated with lower odds of awareness compared with normal waist circumference (AOR: 0.210) (Table 5).

**Table 5:** Multivariable logistic regression model to assess factors associated with awareness and treatment of hypertension For multivariate logistic regression analysis, we took factors that got a p-value of <0.2 in bivariate logistic regression, to see the change in their significance after adjustment. p-value <0.05 was considered significant.

Outcome	Predictor	Category comparison	Adjusted odds ratio	95% CI	p-value
Awareness	Age	≥30 years vs. <30 years	0.336	0.126–0.896	0.029
	Education	Literate vs. illiterate	2.559	1.256–5.211	0.010
	Sitting time	<9 hours/day vs. >9 hours/day	3.611	1.408–9.261	0.008
	Comorbidity	Absent vs. present	0.313	0.136–0.722	0.006
	Waist circumference	Abnormal vs. normal	0.210	0.076–0.582	0.003
Treatment	Education	Literate vs. illiterate	3.934	1.636–9.463	0.002
	Type of family	Joint vs. nuclear	2.715	1.272–5.798	0.010
	Sitting time	<9 hours/day vs. >9 hours/day	3.192	1.231–8.274	0.017

Treatment was significantly associated with education, type of family, sitting time, and comorbidity. Literate participants had higher odds of receiving treatment than illiterate participants (AOR: 3.934). Similarly, participants from joint families (AOR: 2.715) and those with sitting time less than nine hours a day (AOR: 3.192) had higher odds of treatment. In contrast, the absence of comorbidity was associated with lower odds of treatment compared with the presence of comorbidity (AOR: 0.231) (Table 5).

No statistically significant association was observed between the studied variables and control of hypertension after adjustment.

## Discussion

The present study found a hypertension prevalence of 35.7% among adults in a rural area of Delhi, which is comparable to findings from other rural studies in India [11,12]. Although urban populations have historically reported higher prevalence rates, recent evidence indicates a diminishing urban-rural disparity. This shift may be attributed to the rapid urbanization of rural areas, accompanied by lifestyle transitions, dietary changes, and increased availability of calorie-dense packaged foods [13].

Hypertension prevalence was higher among male participants (40.2%) than females (33.9%), a pattern consistent with national and international studies [11,12,14-17]. Additionally, 13% of participants aged below 30 years were found to be hypertensive, corroborating findings from earlier research [8]. The emergence of hypertension at younger ages underscores the expanding burden of the disease and highlights the need for early screening and preventive interventions targeting young adults.

Nearly one-third of the study population was identified as prehypertensive, similar to observations from other rural settings [7]. This finding is of public health importance, as individuals with prehypertension are at a substantially increased risk of progressing to overt hypertension if preventive measures are not instituted in a timely manner.

Although the levels of awareness, treatment, and control of hypertension observed in the present study were relatively higher than those reported in some earlier rural studies, they remain inadequate, particularly with regard to optimal blood pressure control. Female participants showed better awareness, treatment uptake, and control than males, a pattern that has been documented in previous studies [11,12,14,18,19]. The presence of comorbidities was significantly associated with greater awareness of hypertension, which may be explained by more frequent interaction with healthcare services and better health-seeking behaviour among these individuals. Similar observations have been reported in a study conducted in Kathmandu, Nepal [20].

Hypertension is often asymptomatic and may remain undetected for long periods. Historically, the hypertension care cascade has been described using the “rule of halves,” particularly in settings with limited health system access and awareness. In the present study, 61.2% of hypertensive individuals were aware of their condition, 44.8% were receiving treatment, and only 14.6% had controlled blood pressure. These findings are comparable to other studies [8] and highlight substantial gaps between awareness, treatment, and effective blood pressure control. Despite relatively higher awareness, optimal management of hypertension remains inadequate.

The greatest attrition in the hypertension care cascade was observed at the level of control, emphasizing the need for focused interventions to improve medication adherence, follow-up, and therapeutic optimization at the primary healthcare level. Strengthening patient education regarding the asymptomatic nature of hypertension and its long-term complications is essential to improve treatment initiation and control.

Lifestyle modification, a cornerstone of non-pharmacological hypertension management, was inadequately practiced in the study population. While a majority reported reduced salt intake, fewer participants reported reductions in fat consumption or engagement in regular physical activity. These findings differ from those reported by Venkatachalam et al. in rural Tamil Nadu, where higher levels of physical activity and lower levels of dietary modification were observed [21]. Such variations may reflect differences in cultural practices, awareness levels, and local health promotion efforts. Overall, these findings indicate that behavioral interventions and health education remain weak components of rural health outreach and require systematic strengthening.

Given the escalating burden of NCDs and their economic implications for resource-constrained health systems, hypertension represents a strategic entry point for integrated NCD prevention. Cost-effective, population-based strategies emphasizing lifestyle modification through multisectoral collaboration, supported by appropriate policies, regulations, and community education, are imperative. Regular evaluation of these interventions at the local level is crucial to inform scalable and sustainable models for nationwide implementation.

Strengths and limitations

This study’s strengths include an adequate sample size and the use of a pre-tested and pilot-validated instrument. Blood pressure measurements were obtained on two separate occasions, enhancing the accuracy of prevalence estimates, and interviews were conducted by a single investigator to minimize interobserver variability. However, the cross-sectional design limits causal inference, and the predominance of female participants, likely due to the timing of data collection, may affect the generalizability of the findings. Additionally, adherence was assessed using self-reported missed doses over the previous 14 days, which may be subject to recall bias and social desirability bias.

## Conclusions

The present study revealed a high burden of hypertension among adults in rural Delhi. Although awareness of hypertension was relatively high, the rates of treatment and control were far from desirable, reflecting the rule of halves commonly observed in hypertension care. Compliance with medication and lifestyle interventions was also poor. Strengthening community screening and primary care services is necessary for early detection, continuous management, and improved health outcomes.

## Appendices

**Table 6:** Questionnaire

Date of Interview______ Contact Number______		
I. Socio-demographic characteristics		
S. No.	Question	Response
1	Name	
2	Gender	1. Male 2. Female 3. Others
3	Age (in completed years)	
4	Religion	1. Hindu 2. Muslim 3. Christian 4. Sikh 5. Others (specify)
5	What is your highest completed educational level?	1. Profession or honours 2. Graduate 3. Intermediate or diploma 4. High school certificate 5. Middle school certificate 6. Primary school certificate 7. Illiterate
6	Occupation	
7	Marital status	1. Never married 2. Married 3. Separated 4. Divorced 5. Widow 6. Other
8	Total household members	
9	How many people older than 18 years, including yourself, live in your household?	
10	Monthly family income - per capita income	______ INR/month
11	Socio-economic status [22]	>9098, 4549-9098, 2729-4548, 1364-2729, <1364
12	Type of family	1. Joint 2. Nuclear 3. Others
13	Have you ever got your blood pressure measured by a doctor or other health worker?	1. Yes 2. No
14	If yes, how long ago did you get your BP measured?	
15	Have you ever been told by a doctor or other health worker that you have raised blood pressure or hypertension?	1. Yes 2. No
16	If yes, when were you told that you have raised BP?	
17	If yes, have you taken any drugs (medication) for raised blood pressure prescribed by a doctor or other health worker?	1. Yes 2. No
18	Did you get diagnosed with HTN when you suffered from COVID?	
19	If yes, what type of medication have you taken? - Duration	1. Allopathic 2. Ayurvedic 3. Siddha 4. Homeopathy 5. Unani 6. None 7. More than one ____years____months____days
20	How many tablets for HTN are you taking?	1, 2, 3, more than 3
21	From which health facility are you taking medicine?	1. Government 2. Private 3. Both
22	If no, reason for not taking medicine	1. Financial constraint 2. Did not have time 3. Did not have any symptoms 4. Health facilities are away 5. Use home remedies 6. Wanted to consult another doctor 7. Did not want to get dependent on the drugs 8. Heard about the side effects 9. Others
II. Compliance		
23	Do you take medicine as prescribed by the doctor?	1. Yes 2. No
24	Do you usually forget to take your medicine?	1. Yes 2. No
25	In the last 14 days how many times did you miss taking your medication? - Reason	_____days - _____
26	Do you miss taking your medicine because of financial reasons?	1. Yes 2. No
27	Do you sometimes not feel like to take medicine?	1. Yes 2. No
28	Do you miss taking your medicine because of too many pills?	1. Yes 2. No
29	Do you skip your medicine because of the fear of side effects?	1. Yes 2. No
30	Do you miss taking your medicine because there was no one to accompany you to the health care facility?	1. Yes 2. No
31	Do you miss taking your medicine because health care facilities are too far away?	1. Yes 2. No
32	Do you miss taking your medicine because health care facilities do not have the prescribed medication?	1. Yes 2. No
33	Do you miss taking your medicine because health care facility consumes too much time?	1. Yes 2. No
34	Do you miss taking your medicine because of busy schedule?	1. Yes 2. No
35	Did you decrease your salt consumption	1. Yes 2. No
36	Did you decrease your dietary fat consumption	1. Yes 2. No
37	Did you take part in any form of physical activity	1. Yes 2. No
III. Lifestyle behavior		
38	Does your work involve vigorous-intensity activity that causes large increases in breathing or heart rate (like carrying or lifting heavy loads, digging or construction work) for at least 10 minutes continuously?	1. Yes 2. No
39	In a typical week, on how many days do you do vigorous-intensity activities as part of your work?	____days
40	Does your work involve moderate-intensity activity, that causes small increases in breathing or heart rate such as brisk walking (or carrying light loads) for at least 10 minutes continuously?	1. Yes 2. No
41	In a typical week, on how many days do you do moderate-intensity activities as part of your work?	_____days
42	Do you take part in any physical activities?	______min
43	What type of physical activity do you participate in?	1. Brisk walking 2. Running 3. Swimming 4. Bicycle riding 5. Dancing 6. Weight lifting 7. Others______
44	If yes, then how long do you do physical activity?	______days/week ______hours____min(/day)
45	How much time do you spend sitting or reclining in a day?	____hours____min
46	What means of transportation do you use while going to work?	Bus/Car/Scooter/Bicycle/Walking/Autorickshaw/Others
47	What is your dietary preference?	1. Vegetarian diet 2. Non-vegetarian diet 3. Vegetarian + egg 4. Mixed diet (vegetarian + non-vegetarian)
48	What oil do you use for cooking?	Mainly used… 1. Mustard 2. Sunflower 3. Soyabean 4. Olive Oil 5. Ghee 6. Other
49	What do you use as cooking fuel?	LPG/Wood stove/Kerosene stove/Induction stove/Others
50	How often do you use these items?	Item frequency (in a week): Achaar__ Papad__ Namkeen__ Chips__ Pakora__ Fast food__ Others__
51	How many meals do you take in a day - when do you take last meal of the day?	1 2 3 4 5
52	How often do you add salt or a salty sauce such as soy sauce to your food right before you eat it or as you are eating it?	1. Always 2. Often 3. Sometimes 4. Rarely 5. Never 6. Don't know
53	How much salt or salty sauce do you think you consume?	1. Too much 2. Just the right amount 3. Too little 4. Don't know
54	Do you think that too much salt or salty sauce in your diet could cause a health problem?	1. Yes 2. No
55	Do you currently smoke any tobacco products, such as cigarettes, cigars or pipes?	1. Yes 2. No
56	Do you currently smoke tobacco products daily?	1. Yes 2. No
57	How old were you when you first started smoking?	___years, don’t know
58	Do you remember how long ago it was?	___years, ___months, ___days
59	On average, how many of the following products do you smoke each day/week?	1. Manufactured cigarettes___ 2. Hand-rolled cigarettes___ 3. Pipes full of tobacco___ 4. Cigars, cheroots, cigarillos___ 5. Number of *shisha* sessions___ 6. Other___
60	During the past 12 months, have you tried to stop smoking?	1. Yes 2. No
61	In the past, did you ever smoke any tobacco products?	1. Yes 2. No
62	In the past, did you ever smoke daily?	1. Yes 2. No
63	Do you currently use smokeless tobacco products daily?	1. Yes 2. No
64	In the past, did you ever use smokeless tobacco products such as snuff, chewing tobacco, or betel?	1. Yes 2. No
65	Have you ever consumed any alcohol such as beer, wine, spirits, or other local spirits?	1. Yes 2. No
66	Have you stopped drinking due to health reasons, such as a negative impact on your health or on the advice of your doctor or other health worker?	1. Yes 2. No
67	During the past 12 months, how frequently have you had at least one standard alcoholic drink?	1. Daily, 2. 5-6 days per week, 3. 3-4 days a week, 4. 1-2 days a week, 5. 1-3 days a week, 6. Less than once a month, 7. Never
68	In a typical week, on how many days do you eat fruit?	_____days
69	In a typical week, on how many days do you eat vegetables?	_____days
IV. Past and family history		
70	Is there any past medical history/comorbidity?	1. Diabetes mellitus 2. Tuberculosis 3. Hypo/hyperthyroidism 4. CVA/stroke 5. Chronic respiratory disease 6. Cancer 7. Other
71	Did you suffer from COVID-19?	1. Yes 2. No
72	If yes, did you require oxygen at the time of treatment?	
73	Have you taken COVID-19 vaccination?	1. Yes 2. No
74	If yes, which COVID vaccine have you taken?	1. Covishield 2. Covaxine 3. Others 4. Don’t know
75	If yes, how many doses?	1. 1 2. 2 3. Booster
76	Any family history of	1. Hypertension 2. Diabetes mellitus 3. Hypo/hyperthyroidism 4. CVA 5. Other
77	If yes, relation with the family member	
V. Anthropometry		
	Weight (kg): Height (cm): Waist circumference (cm): Hip circumference (cm): Waist/hip ratio:	
VI. Blood pressure measurement		
On first visit: Date: _____ SBP_1_; DBP_1_; SBP_2_; DBP_2_; SBP_3_; DBP_3_. On second visit, after 1 week: Date: _____ SBP_1_; DBP_1_; SBP_2_; DBP_2_; SBP_3_; DBP_3_		
